# Internal Interfaces in Exfoliated MoS_2_ Exhibit Junction-like Behavior

**DOI:** 10.1021/acsami.5c21803

**Published:** 2026-01-12

**Authors:** Emilia S. W. Russell, Oliver M. Rigby, Mark Heath, Ioannis Leontis, Neil Clarey, Saverio Russo, Monica F. Craciun, Andrew J. Gallant, Iddo Amit

**Affiliations:** † Department of Engineering, 3057 Durham University, Lower Mount Joy, South Road, DH1 3LE Durham, U.K.; ‡ School of Engineering, Physics and Mathematics, 5995 Northumbria University, NE1 8ST Newcastle upon Tyne, U.K.; ¶ Centre for Graphene Science, Department of Physics, 3286 University of Exeter, Stocker Road 6, EX4 4QL Exeter, U.K.; § Centre for Graphene Science, Department of Engineering, University of Exeter, North Park Road, EX4 4QF Exeter, U.K.

**Keywords:** MoS_2_, Heterojunction, Scanning Kelvin Probe Microscopy, Interface Traps, Rectification, Band Gap

## Abstract

Mechanical exfoliation remains a ubiquitous method for material deposition in van der Waals layered semiconductors, despite usually producing terraced structures where the layer count changes across the flake, resulting in variations in the band gap magnitude across the device. While most published studies circumvent this phenomenon using sophisticated fabrication processes, these internal interfaces present a unique opportunity for the realization of engineered quantum building blocks within single-crystalline materials. The electronic structure of internal interfaces in MoS_2_, termed here “quasi-heterojunctions”, is studied using a combination of photoluminescence and Raman spectroscopies, Kelvin probe force microscopy, and macroscopic transport measurements. In the transition between 5 to 2 to 1 layers within a single crystal, heterojunctions form, with band offsets of 22 and 24 meV in the conduction bands of the respective junctions. Variations of bandgap and electron affinity, as well as the formation of line defects, are shown to be the primary cause determining the rectification properties of the two junctions in series. Moreover, the formation of a line defect results in a space-charge region that introduces nonlinear properties to its response (*I*–*V*) curves. Finally, computational reconstruction of the measured surface potential using a finite element Poisson solver enables the determination of detailed electronic structures of the constituent segments and their quasi-heterojunctions.

## Graphical abstract

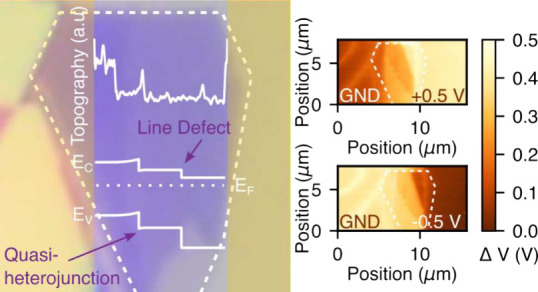

## 1 Introduction

The ability to modulate the bandgap of thin-film molybdenum disulfide (MoS_2_) by both layer variation and strain sets it apart from other transition metal dichalcogenides (TMDCs). It provides opportunities for tunable band gap-based sensing applications and solar cells with enhanced photoresponsivity of up to 350%.^1^ MoS_2_ devices fabricated through mechanical exfoliation from a crystalline source^2^ show superior electrical properties when compared to devices based on lab-grown materials,^3^ due to the large number of defects formed during synthesis. Nevertheless, exfoliated samples show large variability in terms of flake sizes and the number of layers.^4^ The variations in layer-count, which translate to local modulation of the electronic structure (e.g., the magnitude of the band gap and mobility),^5,6^ result in unwanted intraflake interfaces, including energy barriers for conduction and charge trapping centers, which are not well understood.

It is well documented that mobility and resistivity differ between monolayer and bilayer MoS_2_. Baugher et al. found the Hall mobility as a function of carrier density to reach 250 cm^2^/(V s) for the monolayer and 375 cm^2^/(V s) for the bilayer for high densities.^5^ Note that mechanically exfoliated MoS_2_ is typically n-doped on account of sulfur vacancies.^7^ However, reports on the effects of these intraflake interfaces on the macroscopic electrical characteristics and microscopic electronic structure of the interfaces are still limited.

Furthermore, although chemical vapor deposition (CVD) MoS_2_ has been studied extensively for its grain boundaries, which can introduce midgap states, degrade carrier mobility, and alter local optical response,^3,8^ exfoliated MoS_2_ is commonly regarded as single-crystalline with low defect density. In practice, a study published in 2024 used scanning tunneling microscopy (STM) to show that mechanically exfoliated flakes can host grain boundaries, point dislocations, and line defects.^9^ These extended defects can act as scattering centers, recombination sites, and possible leakage pathways in field-effect transistors. However, they remain almost completely uncharacterized compared with point defects such as S vacancies and CVD films.

Taken together, this lack of systematic literature on interlayer interfaces and line defects in exfoliated MoS_2_ highlights a critical gap in understanding the true electronic landscape of these widely used flakes. For electronics and optoelectronic devices that rely on exfoliated materials, both layer junctions and hidden extended defects may play a far greater role than is currently appreciated.

In this work, the formation of an internal heterojunction (termed here “quasi-heterojunction”) in single flakes of mechanically exfoliated MoS_2_ is demonstrated at the boundary between bilayer and monolayer regions. Transport measurements were carried out on devices showing layer count variation such that the current traverses across the quasi-heterojunctions. This highlights the effect of the layer count on the device characteristics. Furthermore, through measurements of work function, photoluminescence (PL), and Raman spectroscopy, a line defect was identified in the monolayer region. Its high-charge trap density was found to influence the rectifying behavior. This highlights that the assumption that mechanically exfoliated flakes are single crystalline and free of extended defects is erroneous and, in turn, could hinder the development of devices made in this way. Finally, the impact of these junctions on the local doping and work function was illustrated using Kelvin probe force microscopy (KPFM) and a Sentaurus DCAD finite element solver.

## 2 Results and Discussion

### 2.1 Optical Microscopy and Atomic Force Microscopy

Figure 1a shows an optical micrograph of a mechanically exfoliated flake on a silicon wafer with a 300 nm thermal oxide layer where three distinct regions of color contrast are visible. Tapping mode atomic force microscopy (AFM) micrographs of the flake were taken (shown in Figure 1b), and the cross section of interest, across the white dotted line, is overlaid as an inset. This confirms a height step difference of 1.8 ± 0.2 nm from the thicker section on the left-hand side to the middle region, which suggests a 3 layer difference, according to reported values of 0.65 nm for single MoS_2_ layers.^10^ AFM was further used to find the step between the middle and right-hand regions to be 0.6 nm. Additionally, the step change between the right-hand side region and the substrate was measured separately and found to be 0.65 nm. It is noted that despite the high contrast seen in Figure 1a, AFM measurements, shown in Figure S1.1, as well as subsequent Raman spectroscopy, confirm that the region is a monolayer. Further details of these measurements are shown in Section S1 of the Supporting Information (SI). Therefore, it is concluded that the device includes layer count transitions from 5 layers on the left-hand side (LHS) through 2 (middle) to 1 layer thick on the right-hand side (RHS), as labeled in Figure 1b as Re1–3.

**Figure 1:**
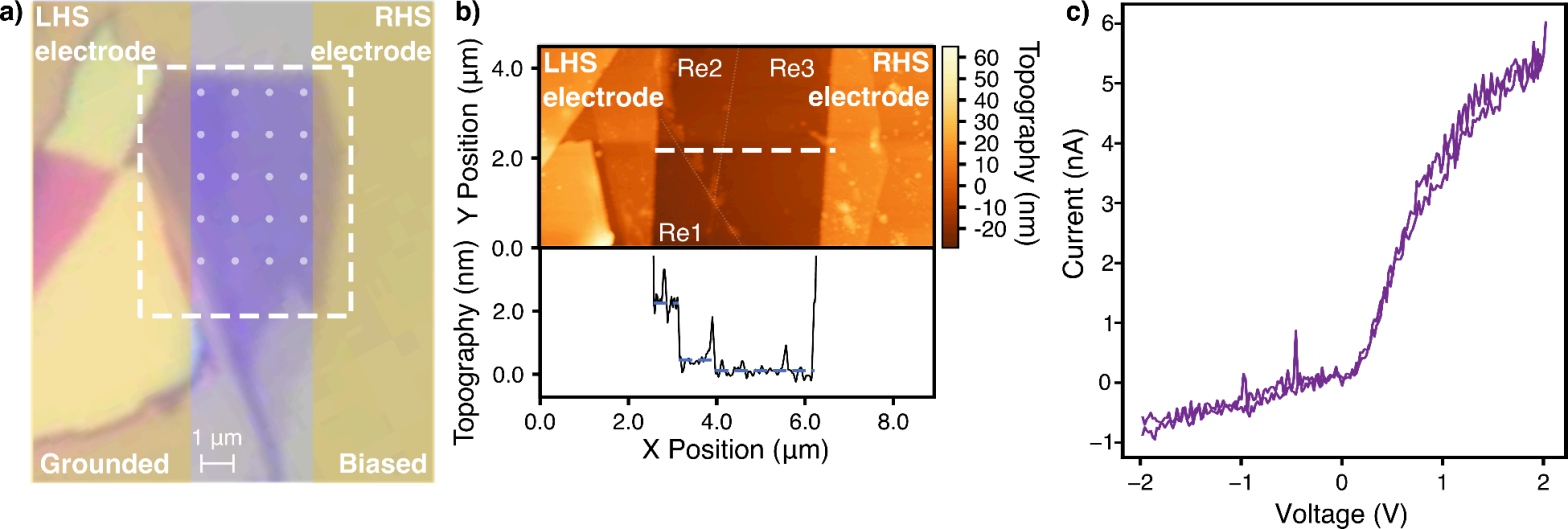
(a) An optical microscope image of the selected flake of mechanically exfoliated MoS_2_. The e-beam-patterned electrodes used for the transport measurements in panel c have been shaded in yellow and labeled, and the dashed white box represents the location of the AFM scan in panel b. The translucent white spots represent the laser locations for the Raman map, discussed later in the paper. (b) AFM micrograph of the layer step region of the flake of interest, with a dashed white line representing the topographical cross section. The inset shows this line scan of the change in layer numbers, measuring 5 layers, 2 layers, then 1 layer of MoS_2_. The labels Re1–3 indicate the three thickness regions (5–2–1 layers) identified along the inset with translucent dotted lines added as a visual guide to the reader along the micrograph layer steps. (c) A current–voltage sweep across the flake of interest, highlighting the partially rectifying behavior, thought to be attributed to the layer change.

### 2.2 Transport Measurements

Electrodes were patterned perpendicular to the optical layer step on the flake by using electron-beam (e-beam) lithography and e-beam evaporation of titanium and gold. The current versus voltage characteristics of the layer-stepped flake are shown in Figure 1c. Crucially, the highly doped substrate was left floating throughout this work to ensure that the electrostatic structure of the interface is altered as little as possible by field effects. The rectifying behavior observed is an indication of an interface where asymmetric modulation of the depletion width causes a favorable direction for the flow of current. This direction aligns with the direction of increasing thicknesses. The opposite polarity, which impedes current flow, corresponds to decreasing layer numbers. This rectifying behavior is typical for heterojunctions. Here, as the layer-count modulation introduces discrete changes in the band gap and thus offsets the bands within a “monolithic” material, the authors term these rectifying interfaces “quasi-heterojunctions”.

Due to the observed nonlinear *I*–*V*, the device will be considered under forward bias when a positive bias is applied to the RHS electrode and in reverse when a negative bias is applied to the RHS electrode.

### 2.3 Raman Spectroscopy

It is important to note here that the underlying assumption of layer-based modulation of the electronic structure suggests that the transition is between regions of different layer counts within the material. This is as opposed to transport within a single layer, decoupled from its neighboring layers. To support this assumption, Raman spectra were collected across the flake, at the sampling points highlighted by the white spot markers in Figure 1a.

It is well-established that the number of MoS_2_ layers can be identified from shifts in the characteristic Raman-active modes. The E_2g_^1^ (in plane vibration of Mo and S atoms) and A_1g_ (out of plane vibration of S atoms) modes were observed around 383 cm^–1^ and 407 cm^–1^, respectively (see Figure 2a), consistent with reported values from previous studies.^11,12^ Upon increasing layer thickness, the E_2g_^1^ mode typically red-shifts while the A_1g_ mode blue-shifts, due to enhanced interlayer van der Waals interactions and dielectric screening effects.^13^ The resulting increase in peak separation *Δω*(E_2g_^1^–A_1g_) is therefore a robust fingerprint of layer number. Finally, while line widths can broaden due to strain, defects, or local disorder, they are also known to reflect the stronger electron–phonon coupling in the direct bandgap monolayer as opposed to the few layers resulting in a broadening of the Raman peaks in monolayers.

**Figure 2:**
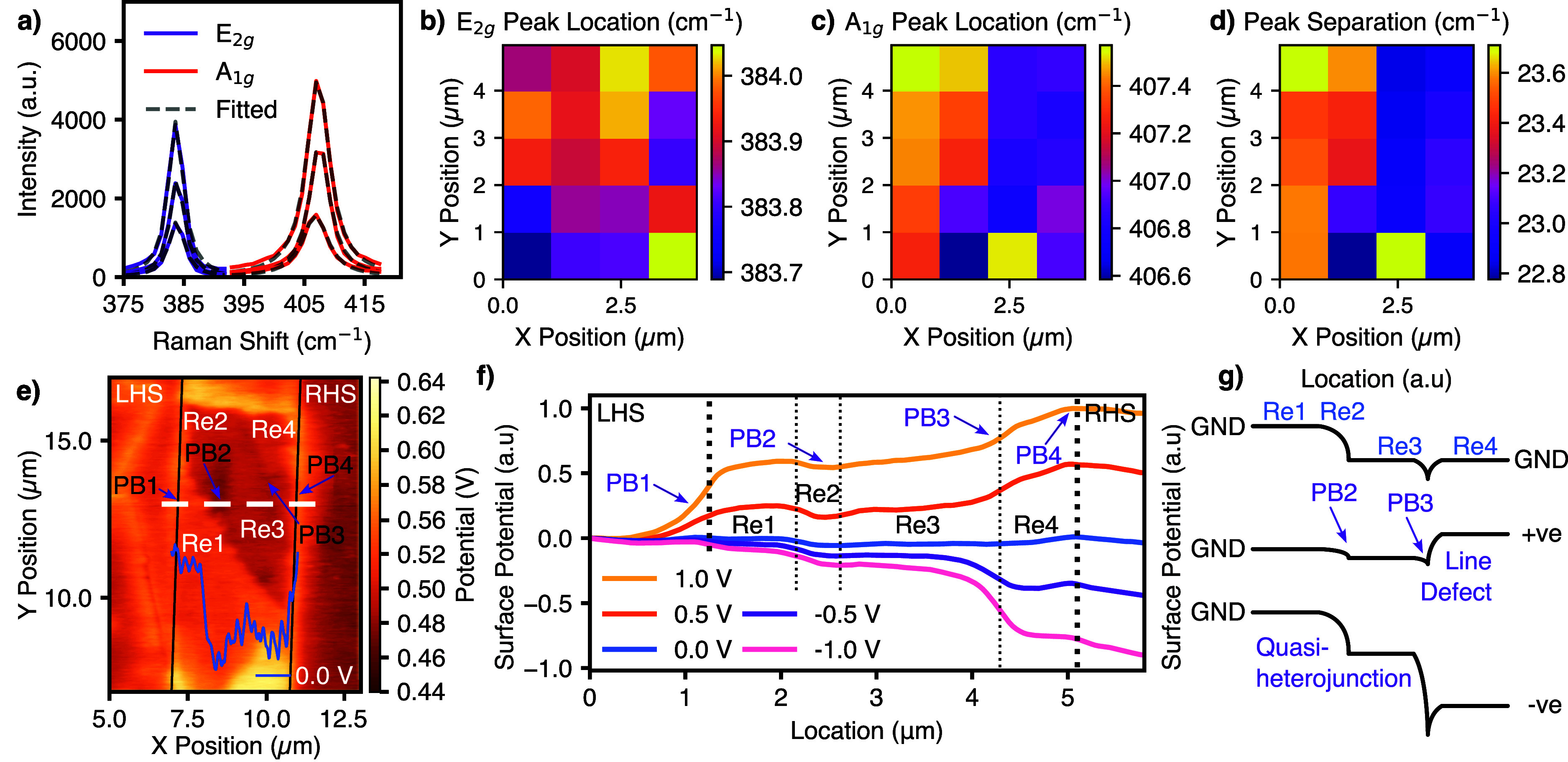
(a) Three spectra from the Raman spectra measured across the flake in intervals of approximately 1 μm between the electrodes and their fitted Lorentzian curves. (b–d) Three Raman maps plotting the location of the peak centers of the curves shown in panel a and their separation. The first map (b) corresponds to the Raman shift of the E_2g_ peak, the second (c) corresponds to the A_1g_ peak, and the third (d) is the difference between the two. Note that there is a clear change in the A_1g_ peak location with the change in flake thickness. (e) A KPFM micrograph showing the surface potential at grounded state (GND), with the inset corresponding to the cross section of interest denoted by the white dashed line. (f) The surface potential across the cross section of interest at different biases. Note the 4 purple arrows highlighting the main potential barriers (PB1–4), between the 3 main flake regions of interest (Re1–3). The first and last potential barriers occurring at the junctions to the electrodes and the middle two occurring at the junction between the layer number change and a line defect, respectively. (g) A schematic surface potential for illustration purposes to discuss the behavior occurring in panels e and f.

To better visualize this change, the peak positions, and subsequent peak separation, at the points across the flake are plotted as color maps and included in Figure 2b–d, respectively. A clear broadening of Raman frequencies can be seen with increasing layer numbers, as was also observed by Velický et al.^14^ who parametrized the broadening Raman mode locations with increasing number of layers of MoS_2_. Therefore, by the large peak separation shown in the Raman color map in Figure 2d, it can be ascertained that the first region is thick, while the third is thinner. The second is too small to accurately characterize with this method and with laser resolution.

Furthermore, while a broadening of Raman peaks can be attributed to strain in the flakes,^15^ the phonon-softening induced red-shift in *only* the A_1g_ peak has been attributed to electron doping,^16^ because of stronger electron–phonon coupling in the A_1g_ mode than the E_2g_ mode. This electron doping could be a result of acceptor-type traps in a grain boundary or line-defect or due to higher n-doping in thinner films of MoS_2_.^7^

To complement the results in Figure 2a–d, the intensity of the two peaks at each point on the Raman map are plotted as two-color maps (included in Figure S2.1 in the SI) where it is clear that the regions of higher Raman intensity correlate to the thicker regions, measured by the AFM. The stronger Raman signal is attributed to the larger geometric thickness of the flake and reinforces the AFM measurements which identified the three different regions of different layer numbers and corresponding different material properties.

### 2.4 Kelvin Probe Force Microscopy

KPFM offers an invaluable tool to understand localized charge transport within a material. Figure 2e shows a KFPM micrograph of the device with both electrodes grounded and with the cross-sectional line scan included as an inset. The surface potential in this micrograph shows several potential regions, aligned with those in the topography, which have been labeled Re1–3. It also shows a number of potential steps or barriers, marked in Figure 2e–g as PB1–PB4.

The darker regions in the KPFM micrographs are areas of higher work functions. At the second potential barrier (PB2) between Re2 and Re3, highlighted by the second purple arrow, a lower surface potential occurs at the step height change between the 2-layer and 1-layer sections. This step height change has been verified by AFM (Figure 1b). This is likely due to a depletion region forming between areas of different bandgaps, as the corresponding different conduction bands shift due to a change in the electron affinity and the formation of a surface charge layer at the quasi-heterojunction. While the main barrier (PB2) appears to be occurring at the step between the 2- and 1-layer regions, a depletion region also forms between the 5- and 2-layer regions. However, the depletion region is larger than the width of the 2-layer section along this cross section of interest. Therefore, it is difficult to isolate which heterojunction is the main contributor to the barrier. The KPFM surface potentials at the 5-layer/1-layer junction and the 2-layer/monolayer junction are included in Figure S3.1 in the SI.

The other contrasting line (marked by PB3 in Figure 2e), represents an additional region of higher work function and coincides with a step height change in topography of 0.1 nm, observed only by the higher sensitivity of the amplitude retrace, which can be thought of as the two-dimensional differential of the topography. As such, it is highly sensitive to step height changes. A magnified version of the amplitude retrace micrograph and line are included in Figure S4.1 of the SI. The change in topography, combined with a corresponding potential barrier, is most likely the result of a line defect, grain boundary, or wrinkle. For clarity, the region *after* the line defect, while materially the same as Re3, has been labeled as Re4.

Figure 2f shows the surface potentials of the same KPFM cross-sectional measurements taken with forward, grounded, and reverse biases applied to the RHS electrode. In the grounded state, there is an overall observed trend of increasing work function with decreasing number of layers. This is contrary to literature observations^17^ of increasing work function with layer number in MoS_2_. However, the authors attribute this change to an improved ability to screen the effects of the substrate and find a critical value of 5 nm, above which the layer number begins to have an impact. This is greater than the thickness of the 5-layer region in this flake. Therefore, the change in the work function observed in this paper is likely due to electron affinity or doping changes.

Under forward bias, there are 6 distinct regions (Res) observed, separated by 4 key potential barriers (PBs), highlighted by the purple arrows. The first barrier is attributed to a Schottky barrier between the LHS electrode and the flake. Assuming that the mechanically exfoliated MoS_2_ flake is n-doped,^7^ the formation of a Schottky barrier confirms the observed surface potential at the grounded state. This shows that the work function of the metal is larger than that of the flake. A detailed discussion of the Schottky characteristics of this potential barrier are included in Section S3.ii in the Supporting Information.

Under reverse bias, the large depletion region formed by the Schottky barrier is expected to be at the RHS electrode because, in the absence of internal depletion regions, the LHS electrode is in forward bias and the RHS electrode is in reverse. Surprisingly, the first significant potential drop occurs at the junction within the monolayer flake section, Re3 and Re4 (PB3, purple arrow 3 in Figure 2). This is indicative of a larger potential barrier, attributed to a larger work function difference between those two sections than between Re4 and the electrode, resulting in a wider depletion region. Crucially, the surface potential difference is observable only when bias is applied. Therefore, it is clear that the barrier is not due to a fundamental material difference between those two regions (such as the work function), which would be observed when grounded. This confirms the earlier suggested theory of a line defect occurring at that junction, which depletes the adjacent areas when under bias depending on the polarity of the potential drop.

The asymmetry driven by the structure of the device is best illustrated using schematic surface potential, as shown in Figure 2g. Surface potential was chosen for the schematic to align with the KPFM micrographs included throughout this paper. For clarity, we note that the surface potential is proportional to the conduction band by a factor of −*q*. For an illustration of the conduction band, including expected carrier action, see Figure S5.1 in the Supporting Information. The model in Figure 2g demonstrates that when a positive bias is applied to the RHS, i.e., the flow of electrons is from left to right, the two main potential barriers to the electron transport (the quasi-heterojunctions and the line defect) are significantly reduced, increasing the efficiency of thermionic or field emission currents. Conversely, when a negative bias is applied, i.e., the flow of electrons is from right to left, the potential induced by the line defect increases and forms the main barrier for current flow. Referring back to Figure 1c, it is clear that the current is suppressed when a negative bias is applied, meaning that the line defect is the main cause of the rectifying behavior.

### 2.5 Photoluminescence Spectroscopy

Photoluminescence (PL) spectroscopy line measurements were taken across the same region of interest measured using KPFM (the PL measurement points are shown as labeled white dots in Figure 3a). Three peaks were observed, corresponding to a bandgap of 1.38, 1.50, and 1.82 eV for the 5-, 2-, and 1-layer sections, respectively, further validating the observations from Raman and AFM. Figure 3b shows the PL spectra of the 5- and 2-layer sections. The peaks measured are aligned with observations from the literature of lower energy bandgaps and weaker peaks, often referred to as “I” peaks,^18^ as they are caused by an exciton in the indirect band gap. Furthermore, this variation in minimum bandgap reinforces the likelihood of the junction between layers acting as a quasi-heterojunction.

**Figure 3:**
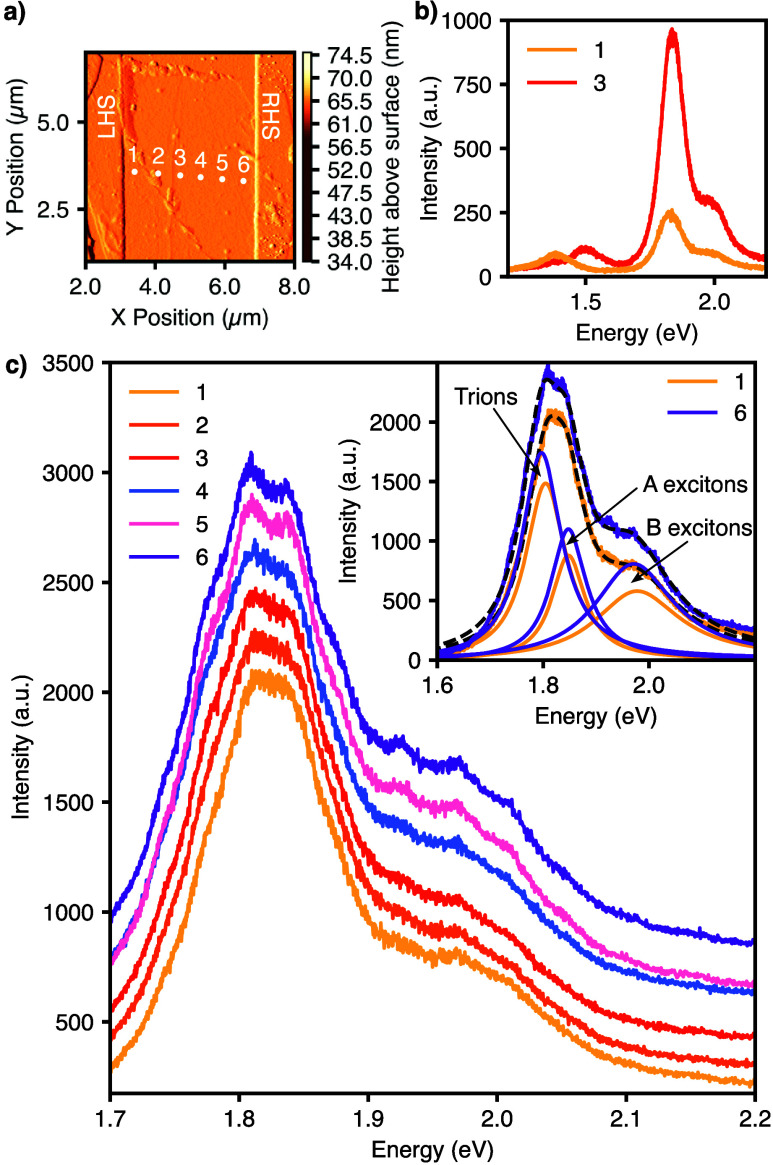
(a) An amplitude retrace micrograph, taken with the AFM, with white dots overlaid to show the PL line scan of 6 points, corresponding to each spectrum in the graph in panel c. (b) Two higher-power PL spectroscopy measurements taken at points 1 and 3 (from white dots in panel a) to detect bandgaps of 5-layer and 2-layer sections. (c) Photoluminescence spectra across the selected flake of mechanically exfoliated MoS_2_ in the locations of the white dots in panel a. Note the “splitting” of the peaks at positions 5 and 6, corresponding to a line defect which can be seen on the amplitude retrace (a), which travels tangentially down from point 5. The inset shows a three-peak deconvolution of spectra 1 and 6 from panel a to identify the exciton and trion contributions to the photoluminescence.

The existence of trapped charges and a line defect were confirmed through PL spectroscopy along the entire line scan, with the results shown in Figure 3c. A red-shift in the primary (direct) bandgap peak was observed as the layer number decreases (values shown in Figure S6.1 of the SI). This peak is ordinarily dominated by the A exciton peak, and a shift in the A exciton is usually associated with increased tensile or decreased compressive strain, similar to strain-induced bandgap shifts reported in ref 19. However, upon the application of three-peak deconvolution using Lorentzian curves on spectra 1 and 6 (shown in the Figure 3c inset), the A exciton, B exciton, and trion contributions^20,21^ can be decoupled and clearly identified.

Therefore, it is clear that the observed split and shift in the primary 1.8 eV peak was actually due to a combination of two mechanisms: (i) a trion shift, which has been linked to increased n-type doping^21^ and an increase in acceptor traps, and (ii) an increase in the magnitude of the exciton peaks, which has been linked to trion dissociation due to adsorbed oxygen at sulfur vacancies and defects sites.^22^ Furthermore, PL redshifts at grain boundaries are commonly observed in CVD grown flakes.^23^ This confirms the observation of a line defect/grain boundary in both the AFM amplitude and KPFM micrographs at PB3. The absence of peak splitting in thicker regions, where a different potential barrier (PB2) is observed, further suggests that the barrier there originates from a quasi-heterojunction at the layer number change rather than from a second line defect.

Recently, red-shifts in both the E_2g_^1^ Raman peak and PL peak were observed in close proximity to the electrodes and were attributed to strain induced in the fabrication process.^24^ It is noted, however, that the device in this work does not exhibit similar behavior, most likely due to fundamental structural differences between mechanically exfoliated and CVD grown flakes.

Additional elements of note in Figure 3c are the additional peaks around 1.78, 1.92, and 1.97 eV, which can occur as a result of charge depletion from increased oxygen adsorption by defects.^22^ This further increases the validity of a line defect being the phenomenon occurring here.

In summary, from the PL spectra in Figure 3, the bandgap of the monolayer section was found to be 1.82 eV. This is consistent with literature values of monolayer MoS_2_. The bandgap of the 5-layer section was found to be 1.38 eV from the secondary peak observed in the PL measurements in Figure 3b, which also aligns with values found in the literature for the indirect bandgap,^25^ and the bandgap of the 2-layer region was found to be 1.50 eV from the PL spectroscopy, which is also consistent with the literature.^26^

## 3 Numerical Model

### 3.1 Model Design

The complexity and interdependence of contributing factors affecting the measured surface potential mean that deconvolution of the underlying physical system can be achieved only through numerical modeling. Therefore, the surface potential was fitted to a model built in the Sentaurus TCAD Device Simulator (Synopsys Inc.) (model shown in Figure 4a and the result overlaid with KPFM measured surface potential is shown in Figure 4b) with the best fitting parameters shown in Table 1.

**Figure 4:**
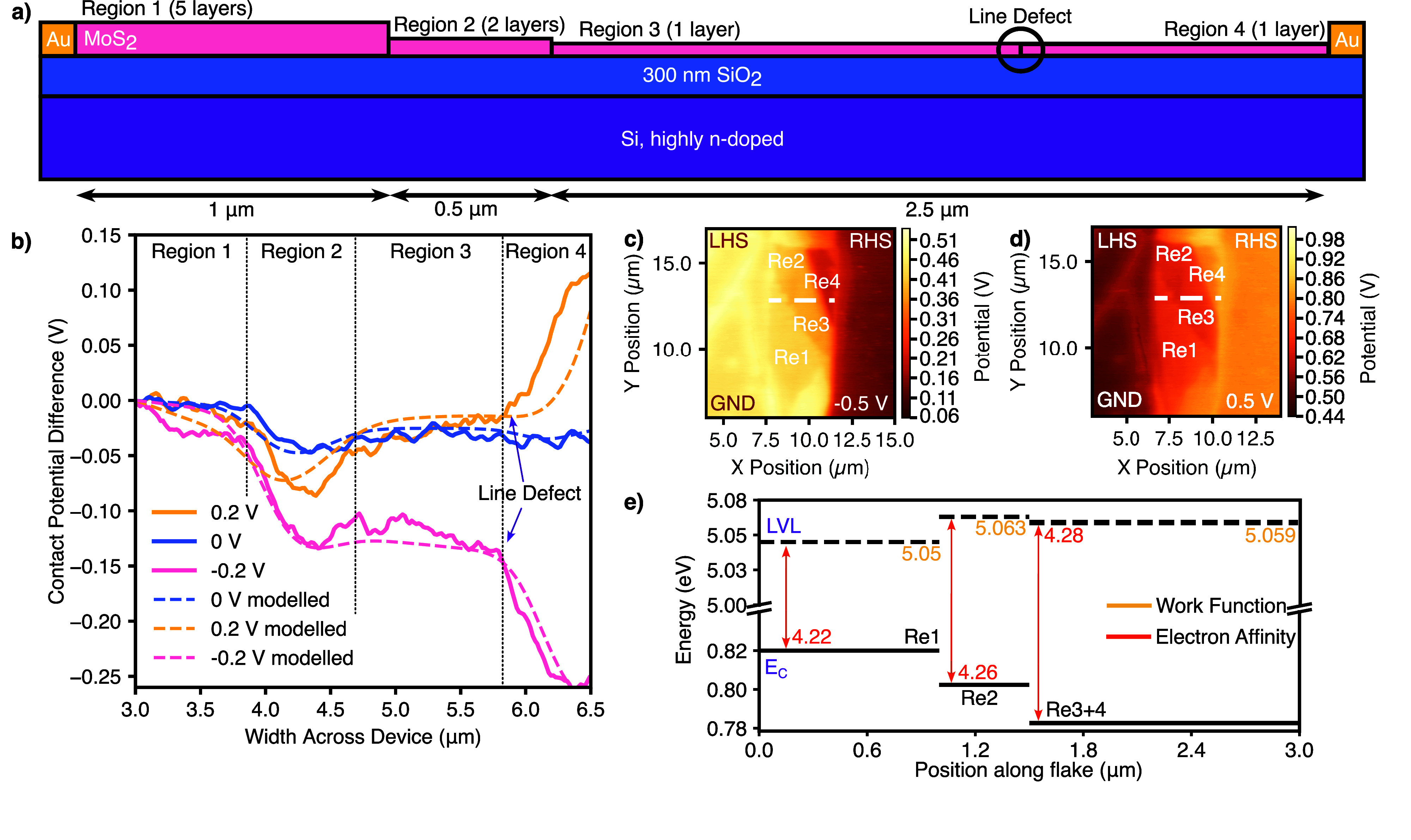
(a) Schematic of the model designed in Sentaurus TCAD Device Simulator. (b) Experimentally measured and modeled surface potential overlaid, where the modeled are represented by dashed lines and KPFM measured by solid lines. (c and d) Corresponding KPFM micrographs under negative and positive 0.5 V bias, respectively, to visualize how the boundaries impact charge transport and result in the partially rectifying behavior. Graphical representation of this same behavior has been included in Figure 2g to help visualize the mechanisms at play. (e) Band diagram with both measured and simulated values to determine the type of heterojunction formed in this device (type II, staggered^27^). The Fermi level, not shown in the figure, is 0 eV.

**Table 1:** Fitting Parameters of the Numerical Model

Region	Re1	Re2	Re3 and Re4
Width [μm]	1.0	0.5	2.5
Electron Affinity [eV]	4.22	4.26	4.28
Doping [cm^–3^]	1 × 10^12^	1 × 10^13^	1 × 10^13^
Interface Traps [cm^–2^eV^–1^]	2.8 × 10^12^		2 × 10^14^ acceptor
			8 × 10^14^ donor
Oxide Traps [cm^–3^]	2.2 × 10^10^	9 × 10^9^	9 × 10^9^

The device was modeled as a 4 μm flake, divided into regions of 5, 2, and 1 layer of MoS_2_, following the AFM measured thickness. Those regions’ widths match those of the cross section of interest from the KPFM measurement, corresponding to 1 μm, 0.5 μm, and 2.5 μm, with a line defect 0.85 μm from the right-hand electrode. Regions 3 and 4 in Figure 4a can be considered as one material. The additional label has been added for consistency with the KPFM figures found elsewhere in this paper. The electrodes on each side provide the boundary conditions to the simulation and are used to apply bias, consistent with the experimental conditions. Finally, a point-spread function was applied to better mimic the “smooth” behavior of the local vacuum level, the main contributor to the KPFM surface potential measurement mechanism.

To start, the bandgaps of the 5-, 2-, and 1-layer sections were set to be 1.38, 1.50, and 1.82 eV, respectively, where the bandgap values were informed by the PL measurements. The electron affinity was set to have an initial value χ = 4.22 eV.^28^ Through successive iteration, the values for electron affinity that gave the best fit to measurements were found to be 4.22, 4.26, and 4.28 eV, for 5, 2, and 1 layer, respectively. Crucially, it is noted that the band gap values are those of the optical band gap, rather than the electronic bandgap; the values are indicative of a trend occurring with thickness; however, the location of the valence band cannot be determined from this analysis. Nevertheless, the TCAD model presented here showed no sensitivity to electronic band gap values, due to sufficiently high n-type doping of the MoS_2_.

The initial n-doping concentration for all of the sections was set to 1 × 10^12^ cm^–3^ based on literature values for the intrinsic carrier concentration of monolayer MoS_2_.^7^ The fitted charge density in Re2 and Re3 was found to be 1 × 10^13^ cm^–3^.

Interface traps were added at the boundaries between Re1 and Re2 and at the line defect (between Re3 and Re4). The best fit trap type and densities at Re1/2 were acceptor traps with exponential distribution from the conduction band, and a concentration of 2.8 × 10^12^ cm^–2^ eV^–1^, with an exponential decay constant of 0.8 eV. The best fit boundary traps at the line defect were acceptor traps with exponential distribution from the conduction band, and a concentration of 2 × 10^14^ cm^–2^ eV^–1^ and an exponential decay factor of 0.12 eV, as well as exponentially distributed donor traps from the valence band, with a concentration of 8 × 10^14^ cm^–2^ eV^–1^.

Oxide traps are a common feature in MoS_2_ on silicon oxide^29–31^ and therefore were included in this model. Initial values in the region of 1 × 10^10^ cm^–3^ acceptor traps were selected and then iteratively trialed to find the best fit to the surface potential results. Interestingly, a higher concentration of oxide traps (2.2 × 10^10^ cm^–3^) worked best for the thicker sections, while 9 × 10^9^ cm^–3^ worked best for the thinner sections.

These parameters are summarized in Table 1.

### 3.2 Converged Model

In order for the work function to increase with decreasing layer number, as shown in Figure 2e, either the electron affinity must be increasing or the Fermi level must be decreasing (as in eq 2). This model found the electron affinity to be increasing with decreasing layer counts, which is in contrast to previous reports where the value of χ was shown to increase with layer count,^32^ which was attributed to quantum confinement effects altering the conduction band minimum (CBM); however, that research was comparing monolayer to 14-layer MoS_2_ and so could have missed nuanced variations occurring between smaller layer steps. Additionally, the paper by Tosun et al.^32^ oversimplifies elements considered in this model, for example assuming invariance in oxide traps and doping between the two thicknesses.

Furthermore, an increase in MoS_2_ electron affinity can be associated with increasing tensile strain,^33,34^ which could be occurring on an atomic level due to the line defect in the middle of the monolayer region.^35,36^ However, as a red-shift was only observed in the A_1g_ Raman peak in Figure 2, an alternative explanation is that the high electron doping causing that red shift is also locally reducing the conduction band energy, as seen by Riley et al.,^37^ resulting in an increase in the local electron affinity.

The greater doping found in Re2 and Re3, relative to Re1, despite a higher work function in these regions, further aligns with the findings from the red-shifted A_1g_ Raman peak. **This indicates that the effect of band discontinuities at the quasi-heterojunction is the dominant factor determining transport in the device.** It is not unexpected that the thinner regions have higher doping than the bulk as there are fewer layers to screen defects;^38^ however, this indicates either that the electron affinity contributes significantly more to the surface potential or that oxide traps are impacting the overall doping profile.

While the line defect trap concentrations may seem high, this was validated by the PL analysis, as the defects initiate charge depletion in the presence of ambient gas (in this case oxygen), which enhances the exciton population dominance from the lower-energy charged trions to the higher-energy, neutral A and B exciton peaks,^22^ as seen in the inset in Figure 3c inset and discussed in Section 2.5.

In addition, the relatively low proportion of interface traps between Re1 and Re2 compared to the line defect confirms the assumption that a “quasi-heterojunction” forming at the boundary is due to the difference in local work functions and bandgaps and not due to a large number of traps, like the line defect.

As these were acceptor traps, they capture electrons from the MoS_2_, reducing the Fermi level and therefore the surface potential, counteracting the change due to electron affinity. Typically, there are more defects in 2D films, so a higher concentration of oxide traps would be expected. However, this is not the case in this study. Instead, it is likely that the thicker layers are passivating fewer traps due to their lower number of defects. Alternatively, it could be that the oxide traps instead represent a different doping distribution in the thicker regions not considered in this model. Another explanation is that the higher conformality that occurs with fewer layers reduces the gaps between the oxide and flake that would otherwise allow for impurity adsorption.

In order for the potential shape to behave as shown in Figure 2e–g and Figure 4b–d, while having the bandgap, work function, electron affinity, and doping profiles found using the Sentaurus model, the heterojunction occurring at the junction between includes a negative band offset in the conduction band, as shown by the band diagram in Figure 4e, where LVL is the local vacuum level and *E*_C,F_ are the conduction band and Fermi level, respectively. The measurements and calculations used to determine the work function and other values are included in SI section S7.

## 4 Conclusion

This paper has uncovered the electronic structure of quasi-heterojunctions formed in the transition regions of differing layer counts and line defects forming within uniform layers as the main mechanism affecting conduction. It further provided evidence that line-defects *do* occur in mechanically exfoliated films and should not be overlooked when characterizing devices. Furthermore, an accurate model was presented quantifying the material characteristics (electron affinity, doping, and optical bandgaps) and trap concentrations contributing to this behavior. Both of these phenomena are common occurrences in devices made using mechanically exfoliated MoS_2_; therefore, this quantification highlights possible challenges in their upscaling as well as opportunities for new devices, for example, single-material rectifying devices.

## 5 Experimental Section

The devices used for this research were fabricated using mechanical exfoliation^39^ of few-layer MoS_2_ flakes from a bulk crystal. These flakes were then transferred onto highly n-doped oxidized silicon wafers (300 nm thermal oxide layer), which had been cleaned using successive dips in acetone, isopropyl alcohol (IPA) and deionized (DI) water, for 10 min each. Optical microscopy contrast analysis^12^ was used to identify flakes with nonuniformity. This included the identification of layer variation and possible line defects. The samples were then coated with poly­(methyl methacrylate) (PMMA) to allow for current injection electrode patterning using e-beam lithography, normal to the observed interfaces. Following development of the pattern, 50 nm gold contacts were deposited by using a 5 nm titanium seed layer to improve adhesion.

The samples were mounted onto Veroboard which allows for simultaneous Kelvin probe microscopy and conductance measurements using wire bonding. Current–voltage measurements were carried out using a Keithley 2450 source measurement unit (SMU). The SMU was remotely controlled using an SCPI-based Python code to automate the transport measurements.

Kelvin probe force microscopy (KPFM) measurements were undertaken using the MFP-3D Origin (Oxford Instruments Asylum Research) atomic force microscope with a nu-nano SPARK 150 Pt conductive AFM tip coated with 40 nm platinum on a 5 nm titanium seed layer. Tapping mode measurements were performed using dual pass scans, where the topography is measured in the first pass and the surface potential is measured on the second pass using a constant tip–sample separation of 5 nm. This reduces the impact of long-range electrostatic interaction between the tip and the sample.^40^

The surface potential, or contact potential difference (CPD), measured by the KPFM represents the difference in work functions between the conductive tip (ϕ_t_) and the sample (ϕ_s_), following

1 $$\mathrm{CPD}=-\frac{\phi_{t}-\phi_{s}}{q}$$

where *q* is the elementary charge.

In addition, the location of the energy levels, based off the work function, can be found using the following equation:^41^

2 $$\phi_{s}=\mathrm{LVL}-E_{F}$$

where LVL represents the local vacuum level and *E*_F_ is the Fermi level, which is determined by other factors such as the density of states and doping concentrations.^41^

Raman and photoluminescence (PL) spectra were measured at room temperature using a Horiba LabRAM Soleil Raman Microscope with a 532 nm laser at 0.27 mW power and 1 μm spot size. Single-point Raman spectra are the result of 5 exposures of the sample to the laser for 30 s per exposure. For Raman maps, each point spectrum is the result of 3 exposures of the sample to the laser for 10 s per exposure. For PL spectra, each point spectrum is the result of 3 exposures for 5 s per exposure. Both Raman and PL measurements were taken at a magnification of ×100 with a numerical aperture of 0.9.

PL spectra of MoS_2_ frequently require deconvolution to determine the exciton vs trion contributions toward the peak, as outlined in ref 21. The most prominent features are the spin–orbit split A and B excitons (1.95 and 2.09 eV,^21^ respectively) at the K/K′ points of the Brillouin zone, which are commonly fitted with Lorentzian (or Voigt) line shapes. The trion, which appears at an energy red-shifted from the A exciton (1.86 eV)^21^ due to the additional Coulomb interaction with an extra charge carrier, is frequently modeled with an additional Lorentzian, sometimes modified to account for its asymmetric broadening due to many-body interactions and carrier density effects. In this work, PL spectra are used primarily for qualitative comparison across thickness-dependent regions, and thus, a simplified deconvolution scheme consisting of three Lorentzian functions (assigned to the trion, A, and B exciton) is employed. The deconvolution of overlapping features facilitates observation of changes in peak position, shape, and the relative intensity across the flake.

## Supplementary Material



## Notes

Verification of the flake thickness, Raman maps, analysis of the interfaces, detailed micrographs of the line defect, model material parameter calculations, location of the optical band gap values, and energy values for the heterojunction (PDF)
