# Supplementary material for: Internal Interfaces in Exfoliated MoS2 Exhibit Junction-like Behavior
Source: ACS Appl Mater Interfaces. 2026 Jan 12;18(3):5721–9. doi: 10.1021/acsami.5c21803 (PMC12862765; doi:10.1021/acsami.5c21803)
Supplement: Supplementary file 1 [file am5c21803_si_001.pdf]

Internal Interfaces in Exfoliated MoS_2 Exhibit Junction-Like Behavior

Supporting Information

E.S.W. Russell [1], O.M. Rigby [2], M. Heath [3], I. Leontis [3], N. Clarey [1], S. Russo [3], M.F. Craciun [4], A.J. Gallant [1], I. Amit [1, *]

[1] Department of Engineering, Durham University, Lower Mount Joy, South Road, DH1 3LE, Durham (UK)

[2] School of Engineering, Physics and Mathematics, Northumbria University, NE1 8ST, Newcastle upon Tyne (UK)

[3] Centre for Graphene Science, Department of Physics, University of Exeter, Stocker Road 6, EX4 4QL, Exeter (UK)

[4] Centre for Graphene Science, Department of Engineering, University of Exeter, North Park Road, EX4 4QF, Exeter (UK)

[*] Corresponding Author Email Address: iddo.amit@durham.ac.uk

Contents

S1 Verification of flake thicknesses **2**

S2 Colormaps of Raman peak intensity **2**

S3 Surface potential at the interfaces **3**

S3.i. Surface potential at 5-2 and 2-1 layer interfaces 3

S3.ii. Surface potential at the metal-semiconductor interfaces 3

S4 Magnified amplitude retrace of line defect **4**

S5 A schematic conduction band of the flake **4**

S6 Location of optical bandgap peaks across flake **6**

S7 Energy values for the heterojunction **6**

S1 Verification of flake thicknesses

In order to verify the thicknesses of different regions on the flake, AFM scans were taken parallel to the electrodes. A measurement was taken between the thin region and the wafer to confirm that it was a monolayer. Then, the measurements taken in the main document (Figure 1b) could be used to find the thicknesses relative to the monolayer region.

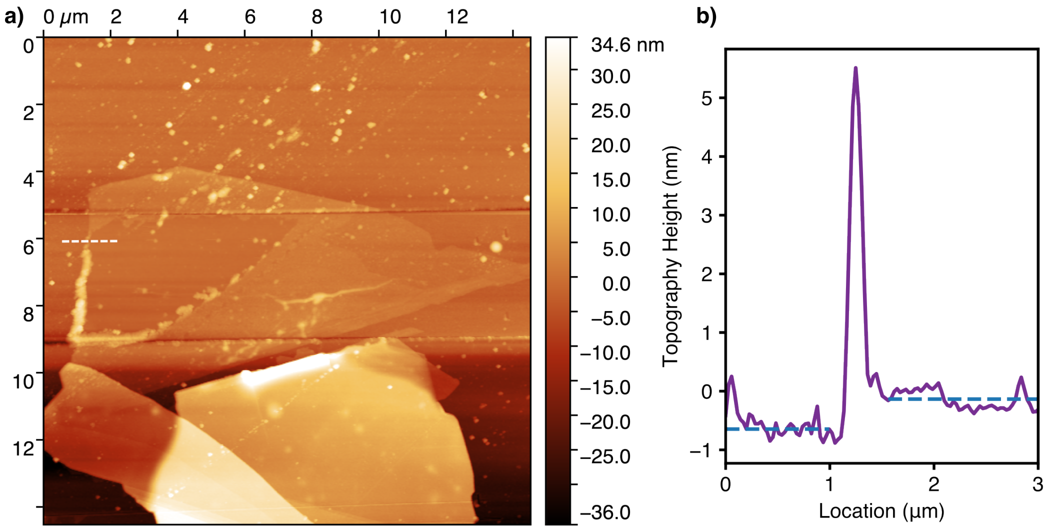

Figure S1.1: a) AFM micrograph of flake, taken parallel to electrodes. b) The line scan from white dashed cross-section included in (a).

S2 Colormaps of Raman peak intensity

After the Lorentzian fitting had been done on the Raman spectra from the map across the flake, the intensities of each peak were converted into a colormap to see the variation across flake thicknesses, shown in Figure S2.1.

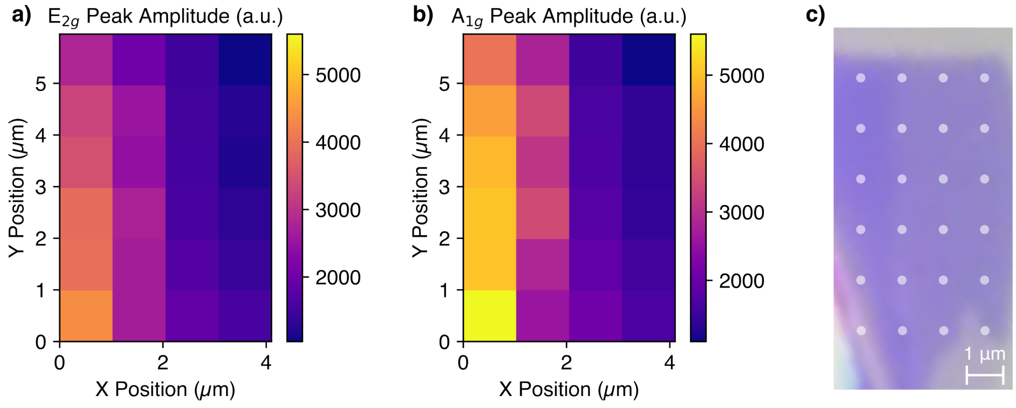

Figure S2.1: a-b) Colormaps of E_{2g} and A_{1g} Raman peak magnitudes, respectively. c) Optical micrograph with white dots indicating locations of Raman mapping in (a-b).

S3 Surface potential at the interfaces

S3.i. Surface potential at 5-2 and 2-1 layer interfaces

In order to examine which layer number change was the predominant contributor to the quasi-heterojunction formation, each independent step (away from the cross-section of interest) was examined and the surface potentials plotted in Figure S3.1.

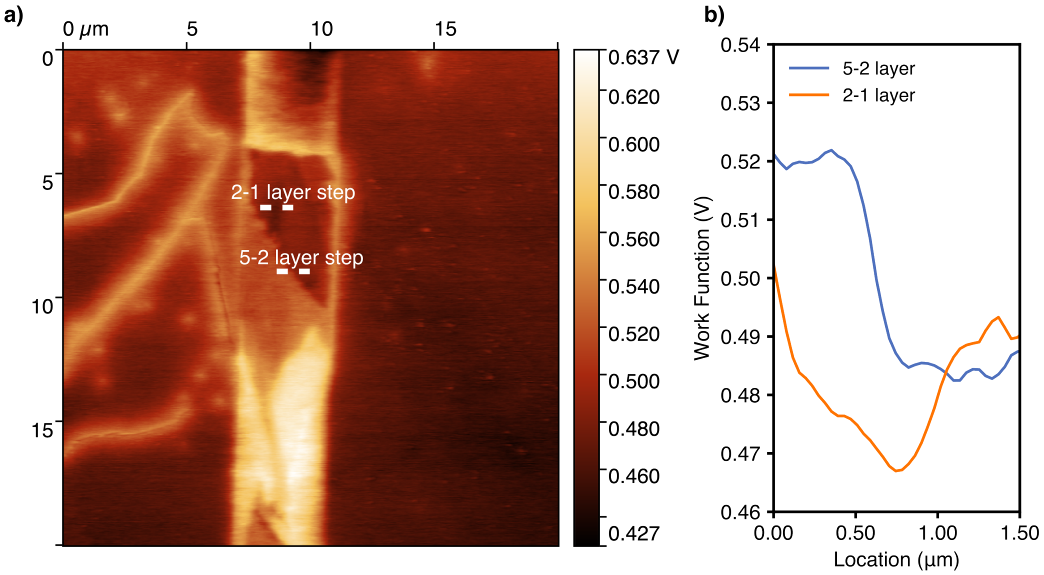

Figure S3.1: a) KPFM micrograph of flake with electrodes grounded. b) Line scan of work function across white dashed cross-sections in (a).

S3.ii. Surface potential at the metal-semiconductor interfaces

The interface between the metal and the semiconductor is likely to create a space charge, which in an n-type semiconductor can be either due to depletion (Schottky contact) or accumulation (ohmic contact). When a Schottky contact is formed, the depletion width is modulated as a square root of the applied voltage:

$$W = \left[\frac{2\varepsilon_0\varepsilon_S}{qN_D}(V_{bi} - V_A)\right]^{1/2} \equiv K \cdot (V_{bi} - V_A)^{1/2} \tag{S1}$$

Where $\varepsilon_0 = 8.85 \times 10^{-14}$ F cm^{-1} is the permittivity of the vacuum, ε_S is the relative permittivity of the semiconductor, q is the elementary charge, N_D is the effective doping, V_{bi} is the built-in potential and V_A is the applied bias using the convention that $V_A > 0$ represents forward biasing. Eq. S1 can be written as a product of a constant, K, that is determined by the material interface system and the effective potential drop, $V_{bi} - V_A$. It is important to note that while the built-in potential is not equal to the Schottky barrier height, Φ_{Bn}, it is related to it via $V_{bi} = \Phi_{Bn} - kT\ln(N_C/N_D)$, where k is the Boltzmann constant, T is the absolute temperature, and N_C is the effective density of states for electrons in the conduction band.

Using Eq. S1 it is possible to **estimate** the value of V_{bi} by tracing the locus of the edge of the depletion region. Figure S3.2 (a) shows the the positively biased and grounded surface potential traces (shown in the main text in Figure 2f), in the immediate vicinity of the LHS electrode. The dashed black line represents the location of the LHS electrode edge, and the dashed gray line is the locus of the inflection points in the surface potential which follow a square-root dependence with applied bias. It is important to note here that the applied bias considered for this calculation is not the bias applied across the entire flake, as that value is divided between several interfaces, but instead it is the potential measured directly after the inflection point. Fitting the data to the simplified version of Eq. S1, the built-in potential is found to be $V_{bi} = 13 \pm 7$ mV. It is important to note here that the inaccuracy of the process for estimating the location of the inflection, which is a result of tip-sample convolution, is the source of the large uncertainty in the reported values.

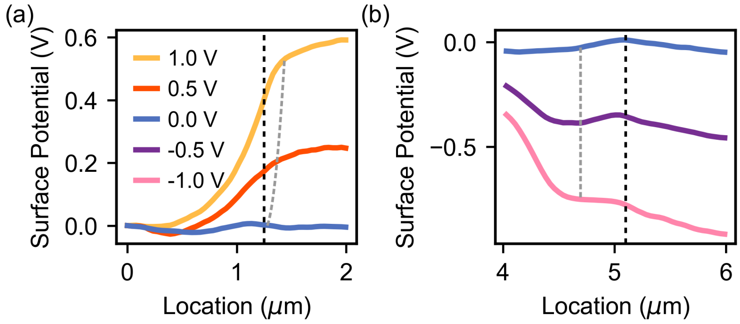

Figure S3.2: Surface potential traces, originally shown in Figure 2f in the main manuscript, shown in details in the vicinity of the LHS (a) and RHS (b) electrodes. The dashed black lines represent the position of the edge of the electrodes. The dashed gray lines represent the locus of the inflection points of the surface potential.

On the RHS electrode, shown in Figure S3.2b, the visually identified inflection point appears at a constant distance from the electrode. It can therefore be concluded that the RHS metal-semiconductor interface is not depleted, *i.e.*, that a Schottky contact is not formed. This suggests that the interfaces formed between the five-layer to metal, and the monolayer to metal are dissimilar, which can also be inferred from the difference in electron affinity and work function seen on both ends.

S4 Magnified amplitude retrace of line defect

Due to the higher sensitivity of amplitude retrace scans to step changes, a magnified micrograph is included here to highlight the visible line defect. Figure S4.1b shows the height change of 0.256 nm.

S5 A schematic conduction band of the flake

To complement the schematic surface potential in the main paper, a similar illustration is included here for visualization of the electron blocking occurring as a result of the line defect when under

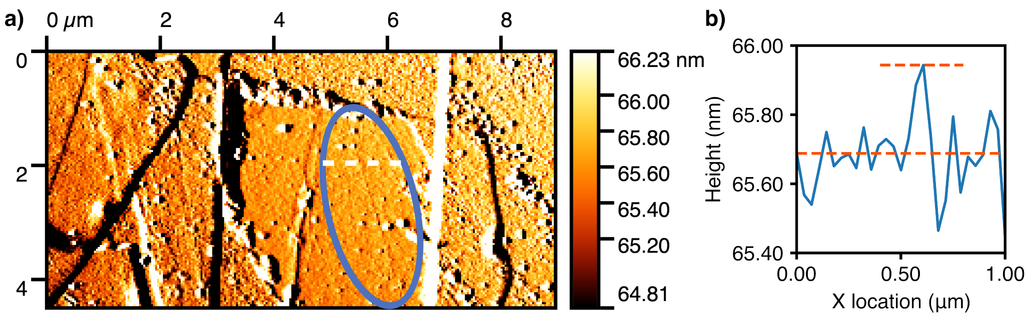

Figure S4.1: a) Magnified amplitude retrace of flake of interest with line-defect highlighted with blue oval. b) Cross section of amplitude retrace region shown with dashed white line in (a). Note the height variation where line defect occurs of 0.256 nm.

negative bias. This behavior is the main contributor to the diode-like response curves, seen in Figure 1c of the main paper.

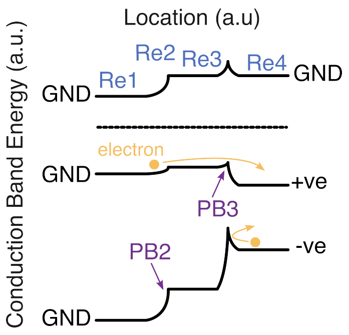

Figure S5.1: A schematic conduction band under varying biases, for illustration purposes, to discuss the rectifying IV behavior observed.

S6 Location of optical bandgap peaks across flake

The energy values for the locations of the optical bandgap peaks for each section of the flake were found using PL spectroscopy and plotted in Figure S6.1

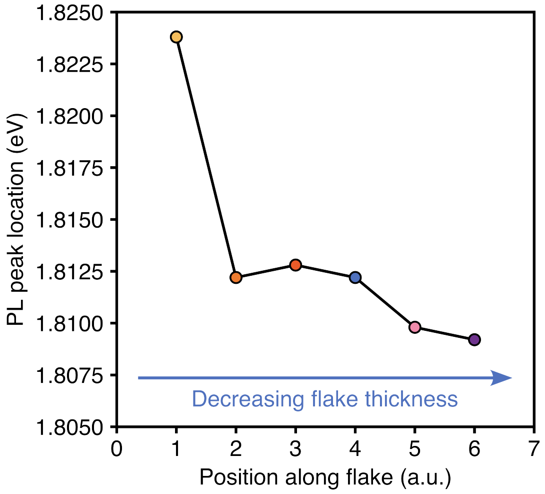

Figure S6.1: Plot of the red-shifting PL line scan peaks as the thickness of the flake decreases. Each point has been colored to match that of the spectra shown in Figure 3c of the main paper for clarity.

S7 Energy values for the heterojunction

Based on the measured value of 0.460 V for the surface potential of gold (shown in Figure S6.1b), and using a reference sample work function of 5.1 eV [1], the work function of the tip can calculated to be 5.560, using equation 1.

Then, using the surface potentials measured in each region, quantified in Table 1, and equation 1 again, the work functions of each region of sample can be calculated. These have been listed in the table, along with the other values found using simulations and PL spectroscopy, all calculated values have been italicized in the table for clarity.

Assuming the material is in equilibrium, and therefore the Fermi levels are aligned, the local vacuum level (LVL) can be considered equal to the work function, using a reference Fermi level at 0 eV.

Once the LVL has been determined, the electron affinity represents the energy difference between the local vacuum level and the conduction band, therefore the conduction band energy can be extracted.

Finally, the valence band energy can be calculated by subtracting the bandgap from the conduction band.

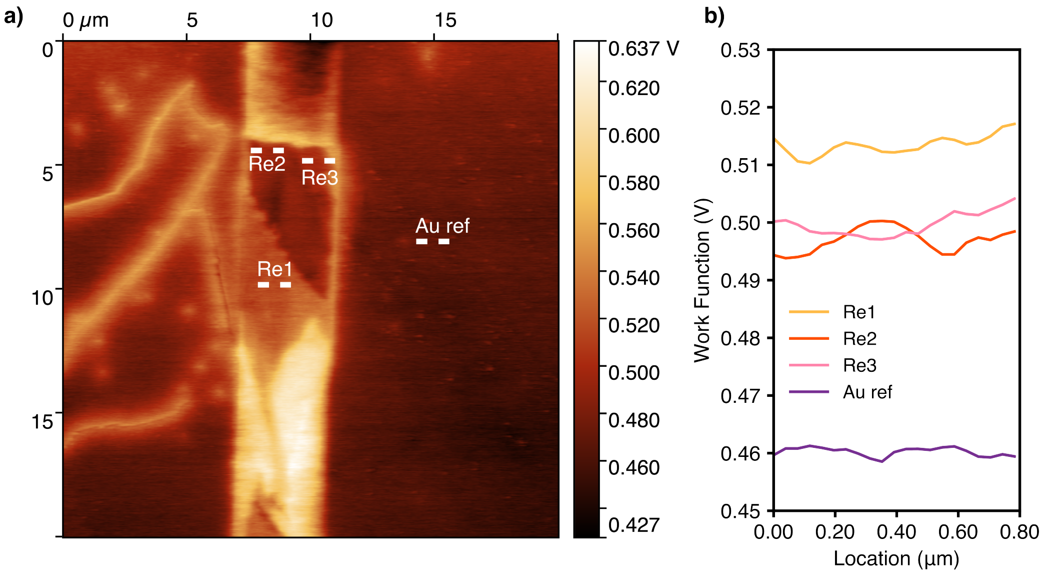

Figure S7.1: a) KPFM micrograph of flake with electrodes grounded, in order to find work functions at each region on the flake. b) Cross sections of KPFM measurements from regions shown with dashed white line in (a).

Table 1: Calculated and measured parameters for band energy diagram.

**Region**	**Re1**	**Re2**	**Re3&4**
**No. layers**	5	2	1
**Width [μm]**	1.0	0.5	2.5
**Surface Potential [V]**	0.515	0.497	0.501
**Work function [eV]**	*5.045*	*5.063*	*5.059*
**Electron Affinity [eV]**	4.22	4.26	4.28
**E_C [eV]**	*0.825*	*0.803*	*0.779*
**Bandgap [eV]**	1.38	1.5	1.8
**E_V [eV]**	*-0.555*	*-0.697*	*-1.041*

References

[1] Lide, D.R. (ed.), *CRC Handbook of Chemistry and Physics*, 89th ed., CRC Press, Boca Raton, FL, 2008, p. 12–124.
